# Afatinib-Associated Cutaneous Toxicity: A Correlation of Severe Skin Reaction with Dramatic Tumor Response in a Woman with Exon 19 Deletion Positive Non-Small-Cell Lung Cancer

**DOI:** 10.7759/cureus.763

**Published:** 2016-09-01

**Authors:** Lindsay P Osborn, Philip R Cohen

**Affiliations:** 1 Medical College of Georgia; 2 Department of Dermatology, University of California, San Diego

**Keywords:** afatinib, exon 19, egfr mutations in lung adenocarcinoma, cutaneous toxicity, skin, non-small-cell lung cancer, adverse events

## Abstract

Epidermal growth factor receptor (EGFR) inhibitors are biological factors used in the treatment of non-small-cell lung cancers (NSCLC) that are positive for EGFR mutations. Afatinib is one such drug that has been approved for use in this capacity. Cutaneous toxicity is the second most commonly reported adverse event with the use of afatinib. A 39-year-old woman with inoperative right lung adenocarcinoma was initially treated with afatinib. She not only developed a severe papulopustular eruption but also had a dramatic reduction of her tumor. Her cutaneous symptoms and lesions were effectively treated with oral and topical corticosteroids, oral antibiotics, and oral antihistamines. After one month of afatinib treatment, her tumor was resected, and there was no evidence of metastases. Afatinib-induced cutaneous toxicity has a positive correlation with tumor response to anti-neoplastic therapy. Supplemental systemic and topical treatments can be initiated to palliate adverse skin events in order to enable adequate duration of treatment with afatinib.

## Introduction

Epidermal growth factor receptor (EGFR) inhibitors are a class of biological agents which act on the ErbB family of tyrosine kinases. Afatinib is an irreversible, multi-receptor inhibitor used for patients with non-small cell lung cancer (NSCLC) who demonstrate EGFR mutations consisting of exon 19 deletion or exon 21 substitution mutation. Due to its irreversible inhibition of multiple ErbB receptors, afatinib offers an option for patients with acquired resistance to the first-generation EGFR reversible inhibitors, namely gefitinib and erlotinib [1-2]. Cutaneous adverse events are more common and severe with afatinib, but afatinib also demonstrates greater progression-free survival when compared with gefitinib [1]. We describe a woman with an inoperable lung cancer demonstrating an exon 19 deletion. She subsequently developed severe cutaneous toxicity associated with dramatic tumor response afatinib, enabling surgical resection of her entire tumor.

## Case presentation

A 39-year-old Caucasian woman presented with a new and persistent cough. Radiographic imaging of her chest demonstrated a mass in the upper lobe of the right lung. Fine-needle aspirate biopsy demonstrated adenocarcinoma. Genomic testing showed the patient’s tumor to be EGFR-mutation positive with exon 19 deletion. She was referred to a comprehensive cancer center for treatment. Her initial computed tomography (CT) scan suggested possible mediastinal invasion at the level of the right brachiocephalic vein. Therefore, she was treated with neoadjuvant afatinib therapy prior to possible surgical intervention. The patient agreed to participate and was explained the nature and objectives of this study, and informed consent was formally obtained. No reference to the patient's identity was made at any stage during data analysis or in the report.

The patient began developing small papules within 24 hours of starting afatinib and by the fourth day of therapy was experiencing severe dermatologic toxicity. Lesions initially appeared on the face, neck, and chest. Individual lesions became confluent and involved 80% of the affected areas. The patient noted her lesions to be extremely pruritic. Her oncologist prescribed oral doxycycline 100 mg twice daily, topical treatment to her skin lesions with clindamycin 1% gel twice daily, and hydrocortisone 2.5% cream.

The patient’s cutaneous symptoms and lesions continued to progress rapidly during the next several days. She was severely debilitated to the point of considering discontinuation of afatinib therapy. Therefore, her oncologist referred her to the dermatology clinic for evaluation and treatment of the drug-associated skin toxicity. After obtaining written consent from the patient, photos of all of the affected areas were taken.

**Figure 1:**
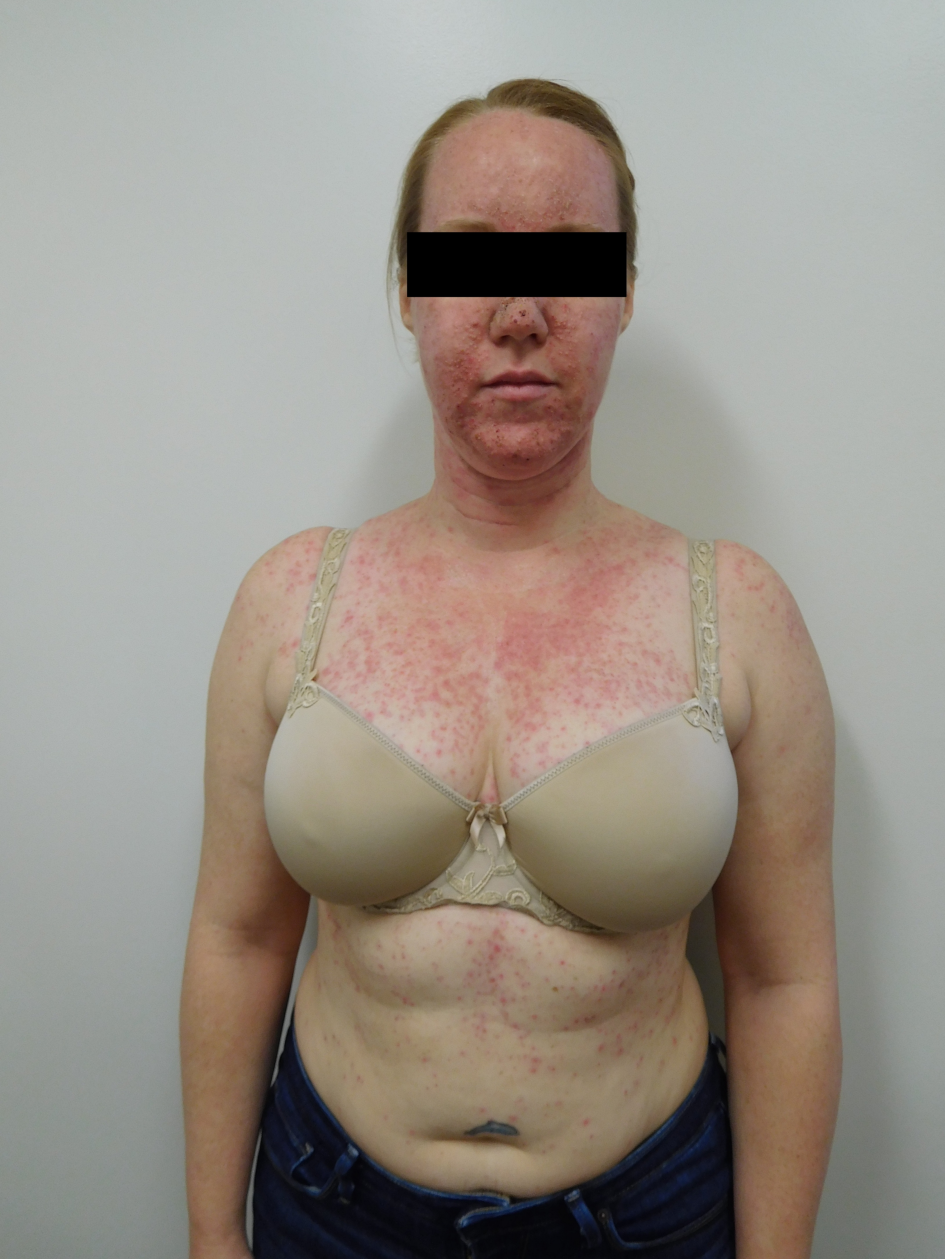
Face and Chest Front view of a 39-year-old woman with NSCLC who developed papulopustular lesions on the face, neck, chest, abdomen, and back after starting treatment with afatinib. Her arms and below her waist were spared.

**Figure 2:**
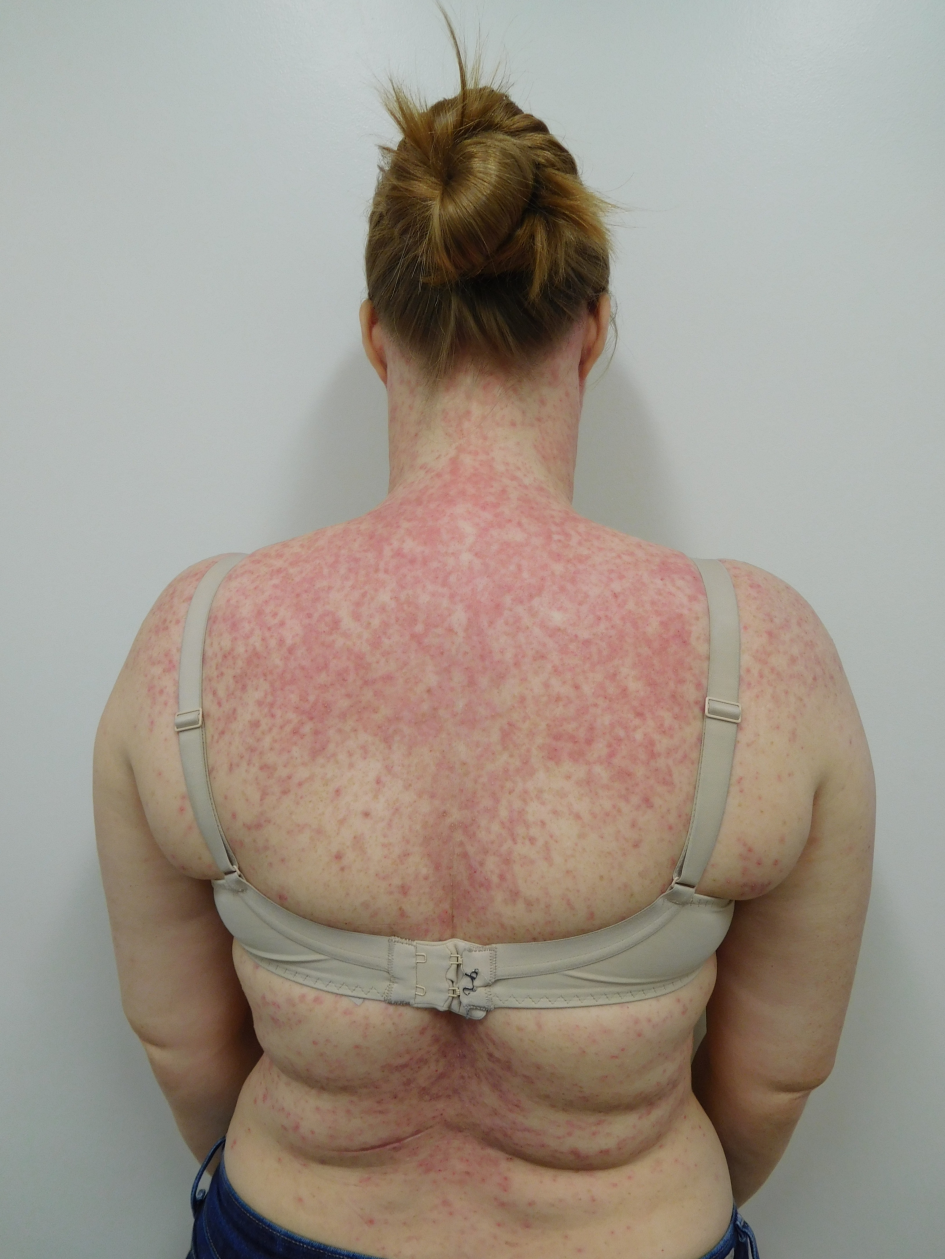
Back

**Figure 3:**
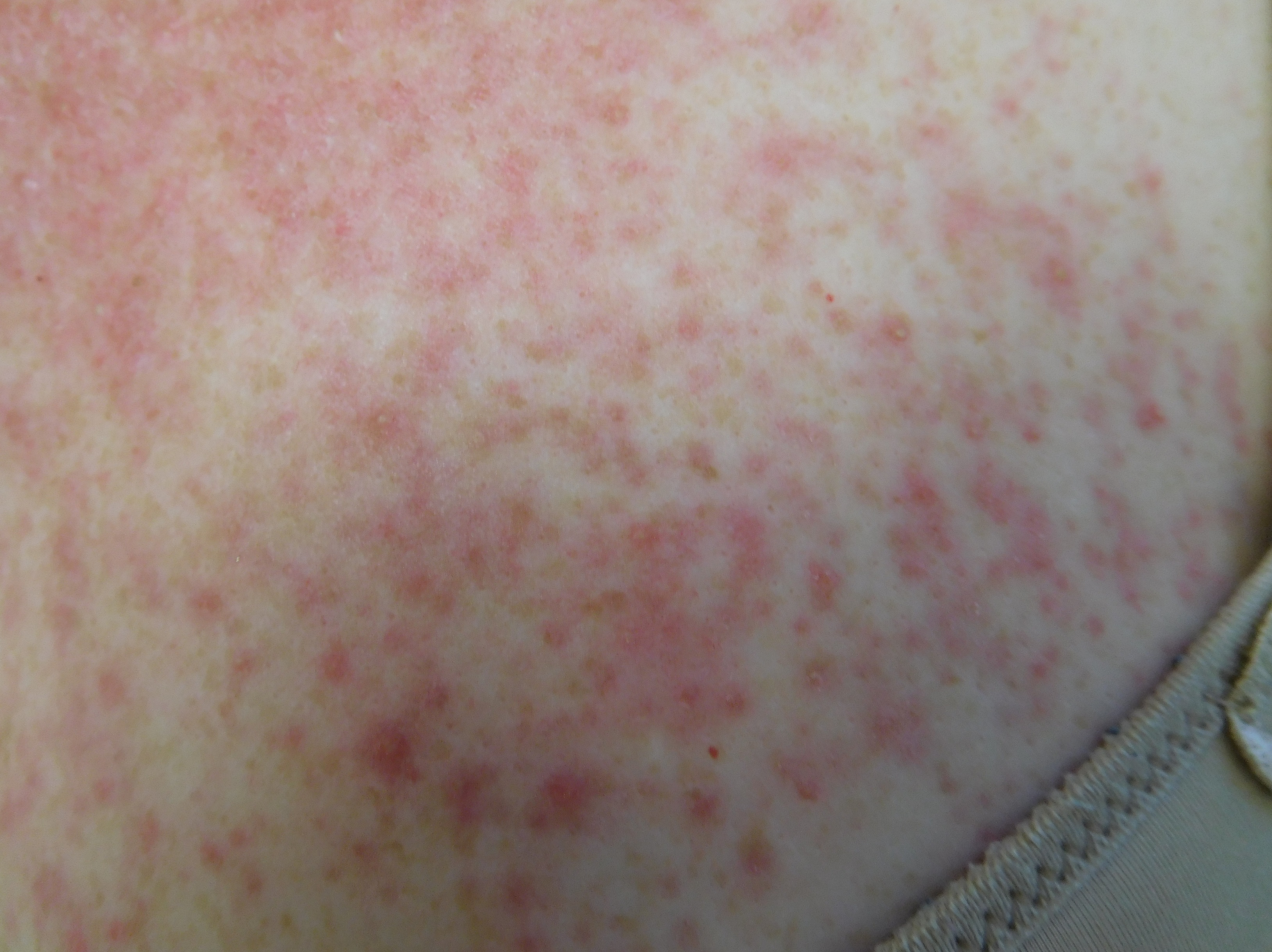
Close-Up of Chest Closer inspection of the chest and back demonstrates numerous pustules on a background of erythema.

**Figure 4:**
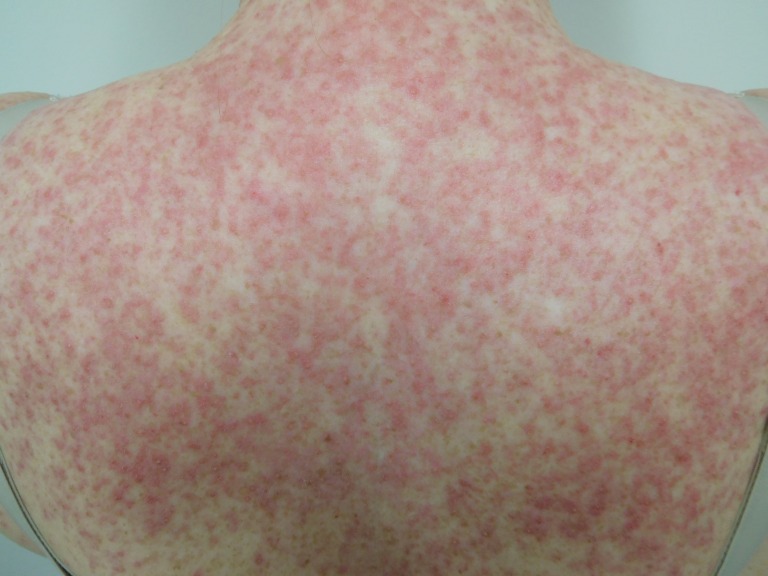
Close-Up of Back

Cutaneous examination after starting afatinib revealed diffuse erythema with individual and confluent papules and pustules on the forehead, face, neck, chest, upper abdomen, and upper back (Figures 1-4).

**Figure 5:**
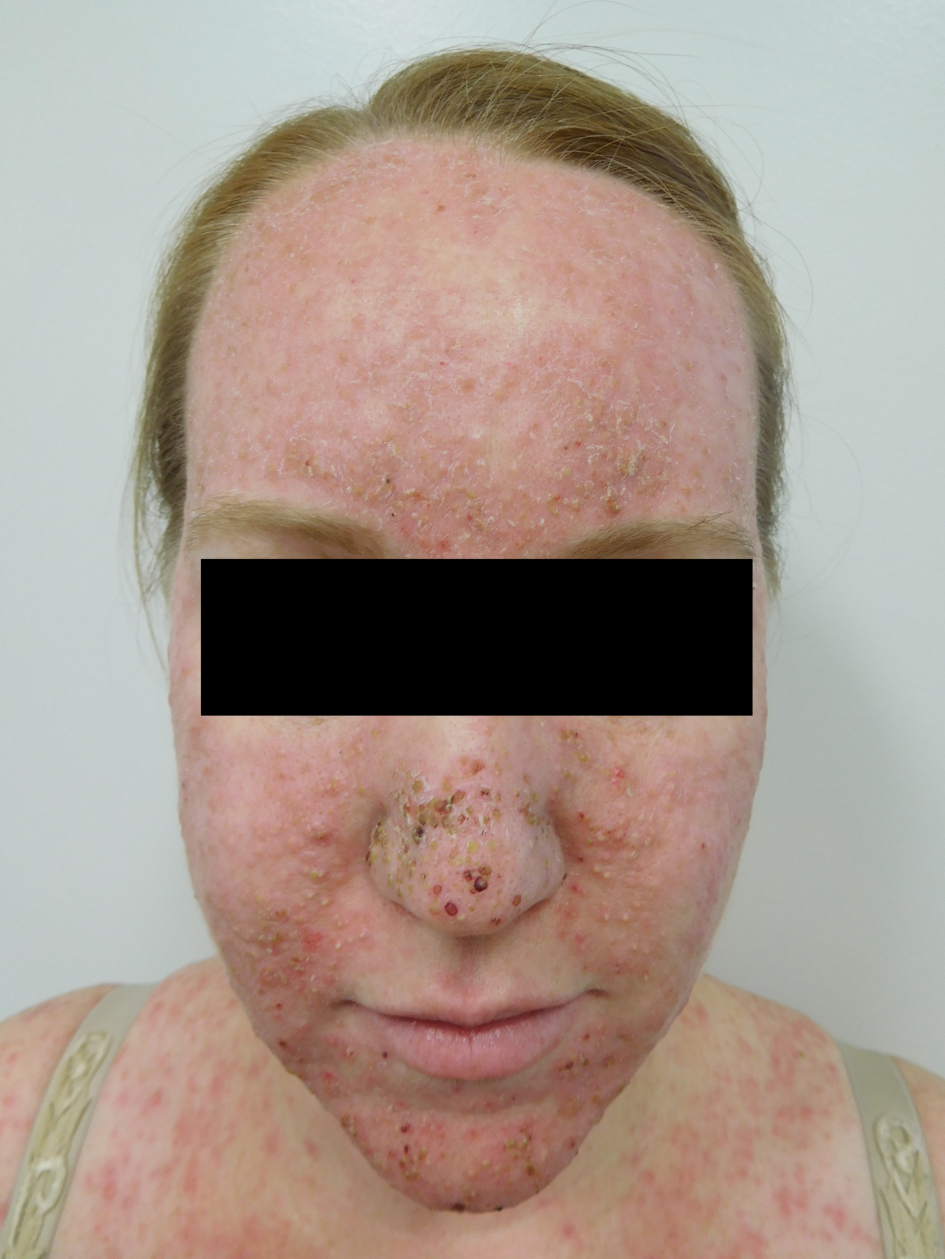
Face Severe acneiform eruption is seen on the face. Crusted papules and pustules involve not only the forehead and nose but also the perioral areas and chin. Papulopustules and scaling on the forehead and scalp are seen.

Her forehead lesions also had superficial scaling, and the lesions on the nose were crusted (Fig 5). There was also diffuse involvement of the scalp. No other hair, nail, or other mucocutaneous lesions were observed.

Therapeutic intervention included oral dexamethasone 4 mg daily for seven days and doxycycline 100 mg twice daily. The clindamycin gel was discontinued since it had elicited severe irritation and pain; topical triamcinolone 0.1% ointment was applied to the face and neck twice daily, and clobetasol propionate 0.05% cream was applied to the chest and back twice daily. To alleviate her pruritis, she received oral fexofenadine 180 mg each morning as well as hydroxyzine 50 mg each evening. During the seven-day course of dexamethasone, she stopped afatinib for three days and then resumed therapy at a 30 mg dose--a 25% reduction from the initial dose of 40 mg.

**Figure 6:**
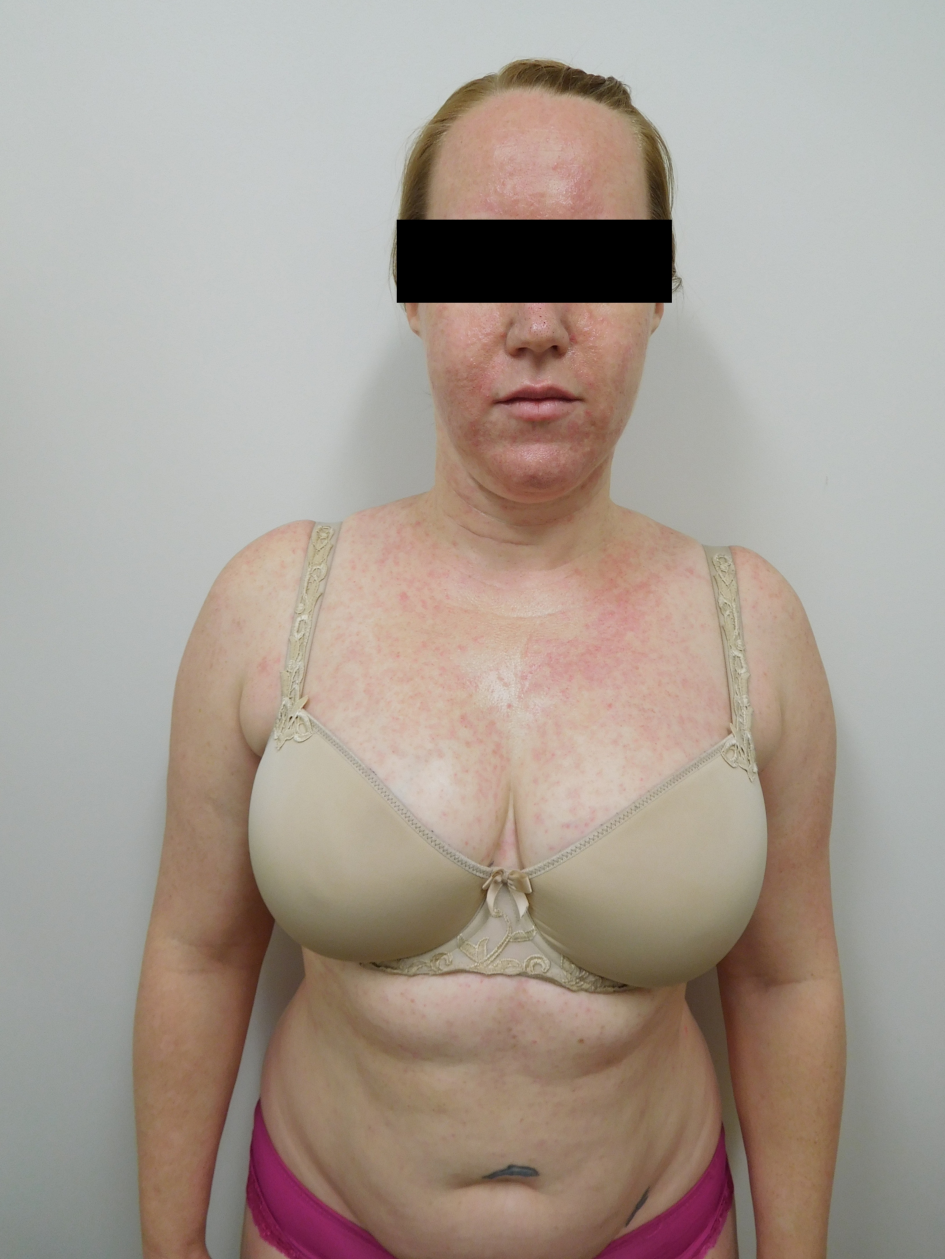
Face and Chest Front view seven days after initiating treatment to ameliorate the cutaneous symptoms and lesions; the papulopustules have flattened, and the associated pain has completely resolved.

**Figure 7:**
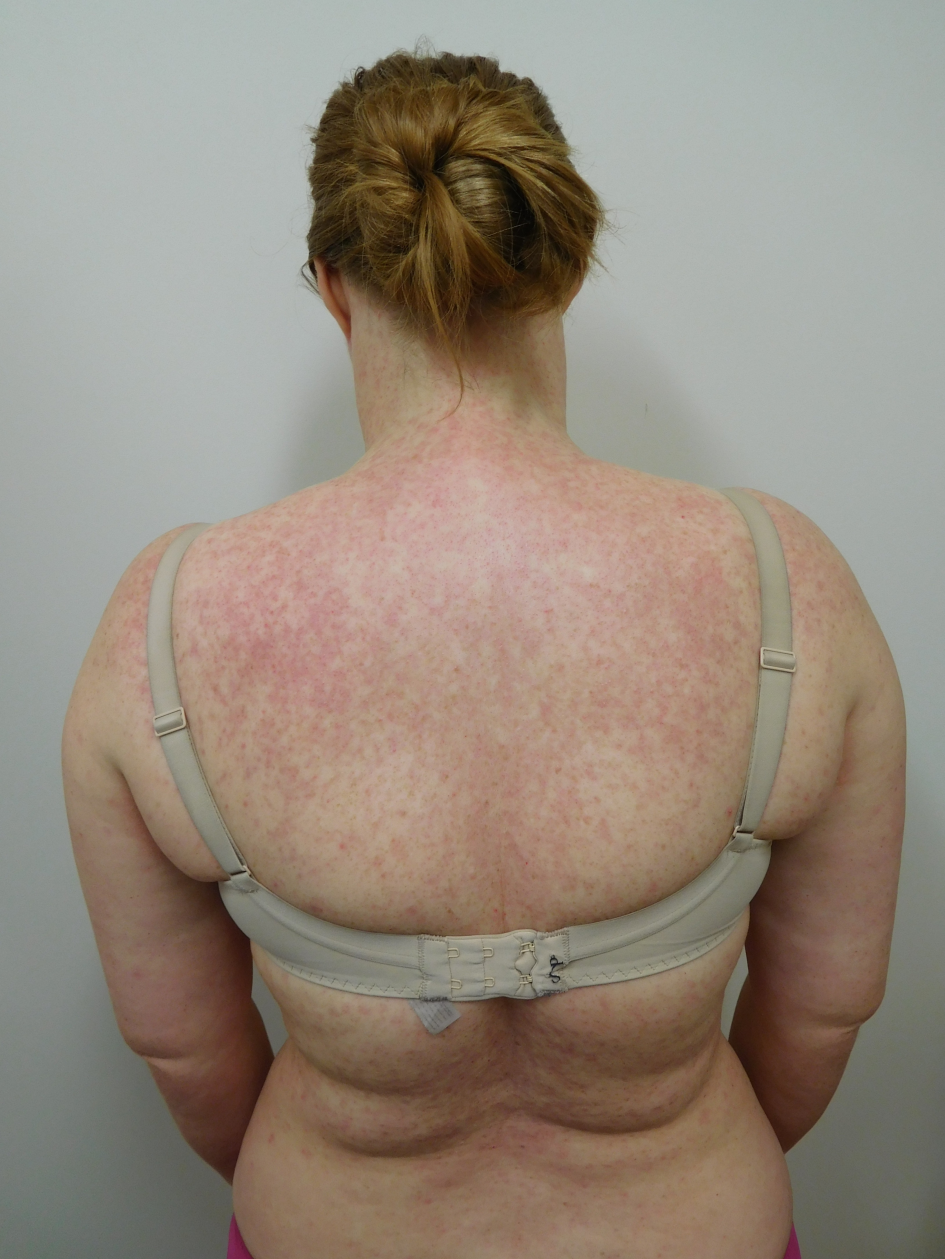
Back Back view seven days after initiation of treatment.

Follow-up examination one week later showed significant improvement in both skin symptoms and lesions. Her skin pain and pruritis had resolved. There were both a resolution of the pustules and a flattening of the papules (Figures 6-7).

**Figure 8:**
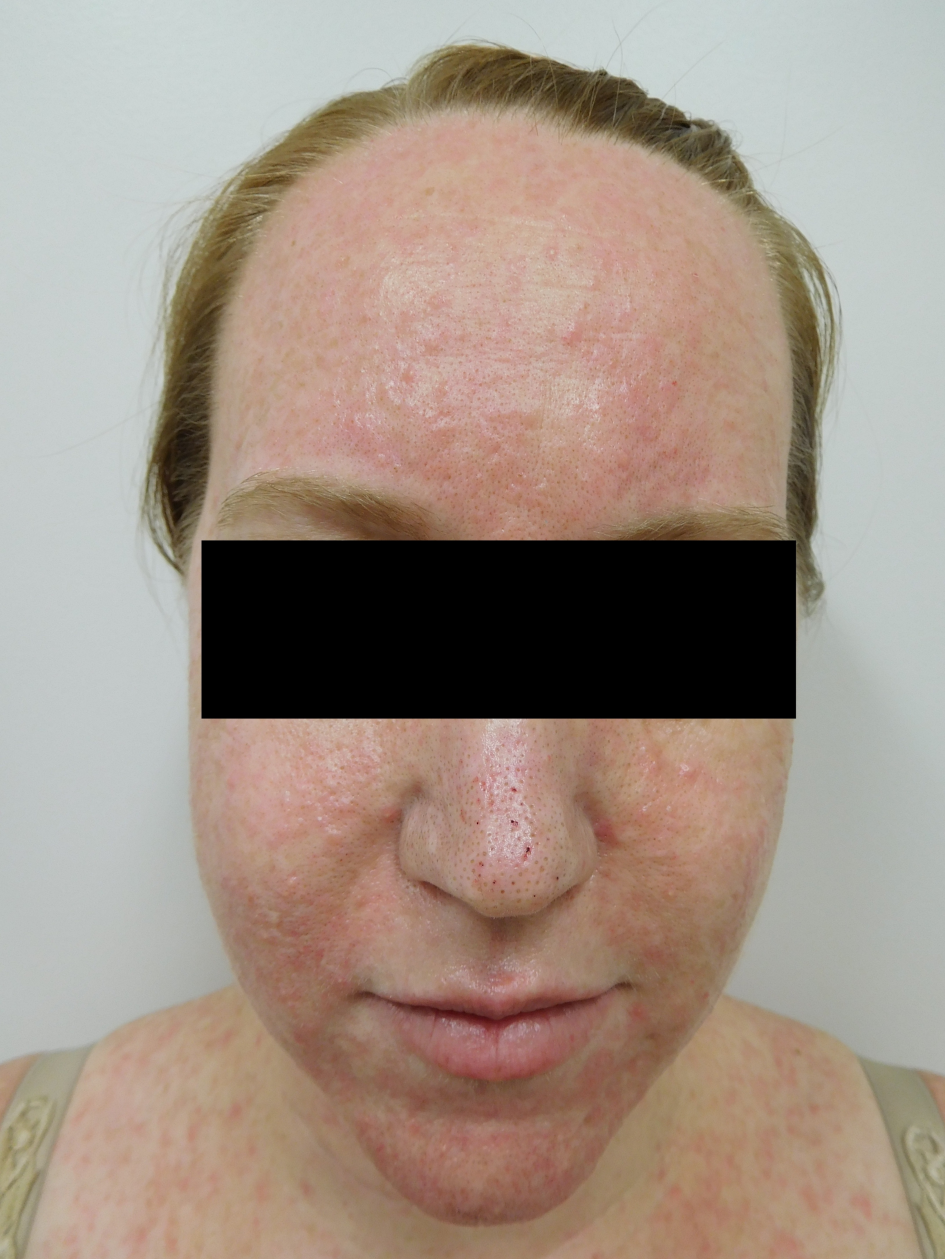
Face A closer view of her face after one week of adjuvant skin treatment shows dramatic improvement of the afatinib-induced cutaneous lesions, particularly on the nose and chin. In addition, the forehead and scalp scaling have resolved.

Erythema persisted on the affected skin areas. Forehead scaling had also resolved, and the crusted lesions on her nose had cleared (Figure 8).

Improvement of the cutaneous toxicity allowed the patient to complete the four-week course of afatinib therapy. A repeat CT scan following the EGFR inhibitor therapy showed a dramatic decrease in the size of the tumor. Additionally, there was no longer any vascular involvement of the right brachiocephalic vein.

Surgical excision of the upper lobe of the right lung and multiple lymph nodes was performed. Pathology demonstrated complete removal of the tumor and showed absence of tumor in any lymph nodes. Within one month of discontinuing afatinib, the patient’s skin lesions completely resolved, and she was able to discontinue all oral and topical therapy.

The patient’s case was reviewed by the cancer center tumor board; the benefit of adjuvant chemotherapy following the successful resection of her tumor was found to be less than 2%. Therefore, the tumor board recommended observation. She will be closely monitored with re-staging every three months.

## Discussion

EGFR inhibitors act by causing dysfunction of the ErbB family of tyrosine kinase receptors. Afatinib specifically acts to inhibit multiple receptors, including ErbB1 (EGFR), ErbB2 (HER2), and ErbB4 (HER4) [3-4]. It does so through covalent binding, thus irreversibly inhibiting these receptors.

EGFR inhibitors have been incorporated into the management of solid tumors. Recently, afatinib was approved for the management of NSCLC. Specifically, afatinib is indicated for use in EGFR-mutation positive patients, including either exon 19 deletion or exon 21 substitution mutation.

EGFR inhibitors are associated with numerous cutaneous adverse events including a papulopustular (acneiform) eruption, paronychia, pruritis, and xerosis [1, 3, 5]. Ranges of cutaneous toxicity from clinical studies of afatinib are shown in Table 1  [9]. Hypertrichosis has also been described in some individuals who have been treated with EGFR inhibitors [6-7].

**Table 1:** Cutaneous Adverse Events Associated with Afatinib [a] [a] Reported ranges from various studies [1, 3, 5, 8]. [b] This classification of drug-associated adverse events includes: Grade 1 (mild symptoms with no intervention indicated), Grade 2 (moderate symptoms with local or noninvasive intervention indicated), and Grade 3 (severe but not life-threatening symptoms; disabling; hospitalization may be necessary) [9]. [c] “Acne” (as cited in the oncology literature) refers to the papulopustular eruption characteristically associated with EGFR inhibitors.

Adverse Event [a]	All grades [b]	Grade 3 [b]
Rash/acne [c]	79-89%	6 - 9%
Paronychia	40-56%	2 - 11%
Pruritis	18-56%	0 - 0.4%
Xerosis	29-33%	0 - 0.4%

There is a direct relationship between the development of skin manifestations of EGFR inhibitors and tumor response to these drugs. The patients who experience more severe cutaneous adverse events have a greater response to the anti-tumor agents. This is also the situation for patients treated with afatinib [8]. At our patient’s initial presentation, she demonstrated an inoperable pulmonary neoplasm due to the suggestion of vascular invasion on her imaging studies. The presence of an exon 19 mutation made her a candidate for neoadjuvant treatment with afatinib. She developed severe cutaneous toxicity that nearly required discontinuation of the therapy. However, successful symptomatic management with oral and topical treatment allowed her to complete the course of afatinib. Indeed, she experienced a marked reduction in the size of her tumor which was subsequently able to be completely excised.

## Conclusions

Afatinib is an EGFR tyrosine kinase inhibitor that acts by irreversible covalent binding to ErbB receptors. It is approved for the treatment of non-small-cell lung cancer in patients who have the exon 19 deletion or exon 21 substitution mutation. Our patient had the exon 19 deletion variety of NSCLC and was treated with afatinib. She developed severe cutaneous toxicity characterized by a diffuse papulopustular eruption on her face, neck, chest, abdomen, and back. She also had a dramatic tumor response to the therapy, thus enabling resection of a previously inoperable neoplasm. In conclusion, similar to other EGFR inhibitors, the severity of a cutaneous reaction to afatinib correlates with the effective response of the tumor to the agent.
